# Exercise-Induced Changes in Enterohepatic Communication Are Linked to Liver Steatosis Resolution

**DOI:** 10.3390/nu17182962

**Published:** 2025-09-15

**Authors:** Yong Zou, Jie Xia, Sen Zhang, Yingjie Guo, Weina Liu, Zhengtang Qi

**Affiliations:** 1 Key Laboratory of Adolescent Health Assessment and Exercise Intervention of Ministry of Education, East China Normal University, Shanghai 200241, China; 52211000009@stu.ecnu.edu.cn (Y.Z.); zhangsen9411@163.com (S.Z.); 2 College of Physical Education and Health, East China Normal University, Shanghai 200241, China; 3 Department of Physical Education, Shanghai Jiao Tong University, Shanghai 200240, China; xiajie@sjtu.edu.cn; 4 Arc Institute, Palo Alto, CA 94304, USA; yingjie.guo@arcinstitute.org

**Keywords:** aerobic exercise, hepatic steatosis, targeted metabolomics, gut microbiota, gut-liver axis, endoplasmic reticulum (ER) stress

## Abstract

**Background/Objectives**: This study aimed to investigate the effects of long-term aerobic exercise on high-fat diet (HFD)-induced hepatic steatosis and its underlying enterohepatic communication mechanisms. **Methods**: C57BL/6J mice were divided into four groups: normal-diet with sedentary (ND-SED), normal-diet with exercise (ND-EXE), HFD with sedentary (HFD-SED), and HFD with exercise (HFD-EXE). After 16 weeks of HFD feeding, ND-EXE and HFD-EXE groups underwent an 8-week aerobic exercise intervention. Hepatic lipid accumulation was assessed via histology and triglyceride (TG) quantification. Liver function and glucose tolerance were evaluated. Gut microbiota composition (16S rRNA sequencing), hepatic bile acid profiles (LC-MS metabolomics), and gene expression were analyzed. **Results**: HFD induced hepatic steatosis, glucose intolerance, and liver injury in mice, all of which were ameliorated by exercise. Compared to HFD-SED mice, which exhibited impaired gut microbiota diversity, exercise restored key genera such as *Faecalibaculum*, and *Turicibacter*. Functional analysis revealed that exercise modulated microbiota shifts in lipid metabolism and secondary bile acid biosynthesis. HFD-EXE mice displayed altered hepatic bile acid composition, characterized by increased tauroursodeoxycholic acid (TUDCA) and reduced taurohyodeoxycholic acid (THDCA). Notably, TUDCA levels correlated with *Turicibacter* abundance, while deoxycholic acid (DCA) was associated with *Faecalibaculum*, independent of precursor availability. Exercise also suppressed hepatic endoplasmic reticulum (ER) stress and downregulated lipogenic genes via the inositol-requiring enzyme 1 alpha (IRE1α)- spliced X-box binding protein 1 (Xbp1s) pathway, while concurrently activating farnesoid X receptor (FXR) signaling to enhance fatty acid oxidation through the FXR-short heterodimer partner (SHP) related to hepatic secondary bile acid abundance change. **Conclusions**: The beneficial effect of long-term aerobic exercise on high-fat diet-induced hepatic steatosis in mice is potentially mediated through structural changes in the gut microbiota, which influence the abundance of hepatic secondary bile acids (TUDCA, DCA) and subsequently regulate the expression of genes involved in lipid metabolism.

## 1. Introduction

Hepatic steatosis, the hallmark pathological feature of metabolic dysfunction-associated fatty liver disease (MAFLD/MASLD), is frequently accompanied by insulin resistance and hepatocellular injury [1]. MAFLD affects approximately 25% of the global population, imposing a substantial economic burden on healthcare systems worldwide [2]. In a 14-year cohort study of 10,500 MAFLD patients, all-cause mortality increased by 93% in middle-aged and elderly individuals following diagnosis [3]. Notably, even mild steatosis alone was associated with a 73% higher mortality risk. Alarmingly, epidemiological forecasts predict a 6–20% surge in MAFLD cases over the coming five years, threatening to overwhelm metabolic disease management systems [4]. While the therapeutic benefits of exercise for metabolic diseases are well-recognized and widely incorporated into standard intervention strategies, the underlying mechanisms remain incompletely understood [5].

Both animal models and human cohort studies reveal that hepatic steatosis is associated with gut microbial dysbiosis, with MAFLD patients exhibiting distinct microbiota composition and metabolic profiles compared to healthy controls [6,7]. Compelling evidence establishes a causal relationship between gut microbial dysbiosis and MAFLD pathogenesis, as demonstrated through fecal microbiota transplantation studies and germ-free animal models [8]. Antibiotic-mediated gut microbiota modulation attenuates hepatic steatosis in preclinical models, demonstrating therapeutically targetable gut-liver axis interactions [9]. Fecal microbiota transplantation from healthy donors reduces hepatic TG accumulation in obese recipients, establishing proof-of-concept for microbiome-targeted metabolic therapy [10]. Patients with metabolic disorders exhibit significant gut microbiota restructuring, characterized by altered Bacteroidetes-to-Firmicutes ratios and depletion of commensal taxa, as revealed by shotgun metagenomic sequencing [11]. Advances in 16S rRNA profiling and shotgun metagenomics have revolutionized microbial ecology, revealing functional roles of gut microbiota that extend far beyond nutrient metabolism—including immunomodulation, neuroendocrine signaling, and xenobiotic processing [12,13]. The gut microbiota generates over 10,000 unique secondary metabolites—far exceeding endogenous human production—that engage in cross-organ communication through endocrine, neural, and immune pathways, effectively constituting a distributed microbial endocrine system [14,15].

Aerobic exercise ameliorates hepatic lipid accumulation and restructures gut microbiota composition, with studies demonstrating its capacity to remodel microbial diversity and taxonomic proportions [16,17,18]. Does exercise remodel gut microbial architecture in mice with hepatic lipid accumulation? If so, are these structural changes functionally associated with exercise-induced attenuation of liver steatosis? Furthermore, through what specific mechanisms might restructured gut microbiota communicate with and regulate hepatic lipid metabolism? To address these questions, we established a murine model of hepatic steatosis and implemented long-term aerobic exercise intervention, aiming to decipher the precise mechanisms by which gut microbiota remodeling mediates exercise’s beneficial effects on liver lipid deposition.

## 2. Materials and Methods

### 2.1. Experimental Animals and Diets

20 Six-week-old male C57BL/6J mice were obtained from East China Normal University’s Experimental Animal Center [License No.: SYXK (Hu) 2019-0019] and housed individually under controlled conditions (22–24 °C, 40–70% humidity, 12-h light/dark cycle) with free access to food and water. Following a 1-week acclimation period, mice were randomly divided into four groups (*n* = 5 per group): normal diet with sedentary (ND-SED), normal diet with exercise (ND-EXE), high-fat diet with sedentary (HFD-SED), and high-fat diet with exercise (HFD-EXE). The high-fat diet (45% kcal from fat, XT310 from Jiangsu Synergetic Bio, Nanjing, China) was administered for 24 weeks, with exercise intervention beginning at week 16 for the HFD-EXE group. The 8-week progressive treadmill training protocol consisted of: 1-week adaptation (10 m/min, 20 min/day, alternate days) followed by 7-week training (starting at 12 m/min for 60 min/day, 5 days/week), including standardized warm-up (5 m/min × 5 min) and cool-down (5 m/min × 5 min) periods. Sedentary controls remained in home cages without treadmill exposure [19]. After experiments, the animals were anesthetized using isoflurane inhalation, followed by blood collection via cardiac puncture. All experimental data points involving animals were included in the statistical analysis. The sample size was determined based on the minimum number required to meet the analytical requirements of the experimental data.

### 2.2. Glucose Tolerance Test

D-glucose (A600219-0005, Sangon Biotech, Shanghai, China) was dissolved in ultrapure water to prepare a 20% (*w*/*v*) solution. Following an 8-h overnight fast (with free access to water) one week prior to sacrifice, mice were subjected to intraperitoneal glucose tolerance tests (GTT) using a SANNO-GA3 glucometer(SANNO, Changsha, China). Briefly, tail tips were clipped to obtain baseline blood samples (time 0), followed by intraperitoneal injection of glucose solution (1 g/kg body weight). Subsequent blood glucose measurements were taken at 15, 30, 45, 60, 90, and 120-min post-injection.

### 2.3. Liver Tissue Sectioning and Staining

Fresh liver tissues were weighed and divided, with 200 mg from the left superior lobe rinsed in PBS and fixed in 4% paraformaldehyde for subsequent paraffin and frozen sectioning. For H&E staining (G1076 kit, Servicebio, Nantong, China), fixed tissues were trimmed, dehydrated through graded alcohols, embedded in paraffin, and sectioned at 4 μm thickness. Sections were floated on 40 °C water, mounted on slides, baked at 60 °C, then stained through sequential immersion in hematoxylin, differentiation solution, bluing reagent, 95% ethanol (with water rinses between steps), and eosin Y, followed by dehydration and neutral resin mounting. For Oil Red O staining, OCT-embedded frozen sections (25 μm) were fixed (G1015, Servicebio), stained with pre-filtered Oil Red O working solution (6:4 saturated dye: water) for 8–10 min at 4 °C, differentiated in 60% isopropanol, counterstained with hematoxylin (3–5 min), blued, and mounted with glycerol gelatin for microscopic evaluation [20].

### 2.4. Hepatic TG Quantification

Approximately 50–100 mg of frozen liver tissue was precisely weighed and homogenized on ice with 900 μL of absolute ethanol (1:9 *w*/*v* ratio). The homogenate was incubated at 65 °C for 15 min to facilitate lipid extraction, followed by centrifugation at 12,000× *g* for 10 min at room temperature. The supernatant was collected for TG quantification using a commercial assay kit (A110-1-1, Nanjing Jiancheng Bioengineering Institute, Nanjing, China) according to the manufacturer’s protocol. Absorbance was measured at 505 nm using a microplate reader, with TG concentrations determined from the standard curve and normalized to tissue weight.

### 2.5. Serum Biochemical Analysis

At the conclusion of the experimental intervention, blood samples were collected via cardiac puncture and transferred to anticoagulant-free tubes. Following 30–60 min of incubation at room temperature for coagulation, samples were centrifuged at 3500 rpm for 10–15 min to obtain serum. Serum alanine aminotransferase (ALT) and aspartate aminotransferase (AST) levels were quantified using commercial assay kits (ALT: C009-3-1; AST: C010-2-1, Nanjing Jiancheng Bioengineering Institute) according to the manufacturers’ protocols.

### 2.6. 16S rRNA Gene Sequencing and Bioinformatics Analysis

Genomic DNA was extracted from samples using either the CTAB or SDS method, followed by assessment of DNA purity and concentration via agarose gel electrophoresis. Qualified DNA samples were diluted to 1 ng/μL with sterile water and amplified by PCR using barcoded primers (515F/806R targeting the V4 region of 16S rRNA) and high-fidelity DNA polymerase (M0532S, New England Biolabs, Boston, MA, USA). PCR products were verified by 2% agarose gel electrophoresis, then pooled in equimolar amounts for gel extraction (28704, Qiagen, Hilden, Germany) of target fragments. Sequencing libraries were prepared using an Illumina kit (20015963, Illumina, San Diego, CA, USA) and quantified by Qubit and qPCR before NovaSeq6000 sequencing.

Raw sequencing data were demultiplexed according to barcode and primer sequences, followed by read assembly using FLASH to generate Raw Tags. Data quality control was performed through: (A) Quality trimming: Raw Tags were truncated at the first base with quality score ≤ 19 in a 3-base window. (B) Length filtering: Tags retaining < 75% of original length after trimming were discarded. Chimeric sequences were removed by comparing against the reference database (SILVA132 SSUrRNA) using Vsearch (version 2.26.0), yielding Effective Tags for subsequent analysis.

Operational Taxonomic Units (OTUs) were clustered at 97% similarity threshold using Uparse, with representative sequences selected based on highest abundance. Taxonomic annotation was performed against the SILVA132 database (threshold 0.8–1) using Mothur (version 1.48.0), generating compositional profiles at seven taxonomic levels (phylum to species). MUSCLE was employed for multiple sequence alignment to determine phylogenetic relationships among OTUs. All samples were rarefied to the minimum sequencing depth for subsequent alpha and beta diversity analyses [21].

### 2.7. Bile Acid Profiling by UHPLC-MS/MS

For each sample, 25 mg of tissue was homogenized in 1.0 mL of pre-chilled (−40 °C) extraction solvent (acetonitrile: methanol: water = 2:2:1, containing 0.1% formic acid and isotope-labeled internal standards) using a three-step protocol: (1) vortexing for 30 s, (2) mechanical homogenization (35 Hz, 4 min), and (3) ice-water bath ultrasonication for 5 min (repeated 3×). After incubation at −40 °C for 1 h, samples were centrifuged (12,000 rpm, 4 °C, 15 min) to collect supernatants for analysis.

Chromatographic separation was achieved on an Agilent 1290 UHPLC system equipped with a Waters ACQUITY UPLC BEH C18 column (150 × 2.1 mm, 1.7 μm) maintained at 60 °C. The mobile phase consisted of (A) 1 mmol/L ammonium acetate + 1 mmol/L acetic acid in water and (B) acetonitrile, with a 1 μL injection volume. Mass spectrometric detection was performed using a Thermo Q Exactive Focus instrument operating in polarity-switching mode (+3500/−3100 V spray voltage) with optimized parameters: sheath gas (40 arb), auxiliary gas (15 arb), auxiliary temperature (350 °C), and capillary temperature (320 °C). Calibration standards (10 mmol/L stock) were serially diluted with constant internal standard concentrations. The method was validated with the following performance characteristics: (A) Linear range: R^2^ > 0.9954 (1/× weighted least squares regression). (B) Sensitivity: LOD 0.49–15.62 nmol/L; LOQ 0.98–31.25 nmol/L. (C) Precision: RSD < 5.3% (*n* = 3 QC replicates). (D) Accuracy: 102.0–113.3% recovery. This optimized PRM-based workflow enabled sensitive and accurate quantification of bile acids in biological matrices [21].

### 2.8. Quantitative Real-Time PCR (qPCR) Analysis

Liver tissue samples (20 mg) were homogenized, and total RNA was extracted using Beyotime Biotechnology RNA extraction kit according to the manufacturer’s protocol. RNA purity and concentration were determined spectrophotometrically, followed by dilution with DEPC-treated water to normalize concentrations across samples. Reverse transcription was performed using Hifair^®^ III Reverse Transcriptase (11141ES60, Yeasen Biotechnology, Shanghai, China) under standard conditions. Quantitative PCR was carried out on a QuantStudio 5 Real-Time PCR System (Applied Biosystems, Foster City, CA, USA) using SYBR Green master mix (11202ES03, Yeasen Biotechnology, Shanghai, China), with each sample run in technical triplicates. Relative gene expression was calculated by the ΔΔCt method using appropriate reference genes. All primer sequences were obtained from PrimerBank (https://pga.mgh.harvard.edu/primerbank/, accessed on 21 August 2024) and validated their specificity from UCSC In Silico (http://systemsbiology.cau.edu.cn/cgi-bin/hgPcr, accessed on 21 August 2024).

### 2.9. Microbiome and Statistical Analysis

Microbial diversity analysis was performed using QIIME (v1.9.1) to calculate alpha diversity indices (Observed-OTUs, Chao1, Shannon, and Simpson), with species accumulation curves generated in R (v4.3.2). Alpha diversity comparisons between groups were conducted using both parametric (*t*-test/Tukey’s test) and non-parametric (Wilcoxon/Kruskal–Wallis with agricolae package) tests based on group numbers. Beta diversity was assessed through Unifrac distance metrics and UPGMA clustering (QIIME), with principal component analysis (PCA) visualized using R’s ade4 and ggplot2 packages (v2.15.3). Differential abundance testing was performed at all taxonomic levels using Metastats with Benjamini–Hochberg FDR correction (q-value), supplemented by ANOSIM (vegan package) for community dissimilarity assessment.

Functional prediction was conducted via PICRUSt2, which: (A) Constructed phylogenetic trees using ASV/OTU tables and GreenGenes/SILVA reference sequences. (B) Inferred metagenomic content through ancestral state reconstruction. (C) Annotated Kyoto Encyclopedia of Genes and Genomes (KEGG) pathways (Levels 1–3), enzyme classifications (EC), and orthologs (KO) [21].

The experimental multi-group data were analyzed using two-way ANOVA, with high-fat diet and exercise as the two factors, and multiple comparisons were adjusted using the Bonferroni method. Comparisons between two groups were performed using the *t*-test. Data are presented as mean ± standard error of the mean (M ± SEM), with statistical significance set at *p* < 0.05.

## 3. Results

### 3.1. Aerobic Exercise Alleviates High-Fat Diet-Induced Hepatic Steatosis and Liver Injury

**Figure 1:**
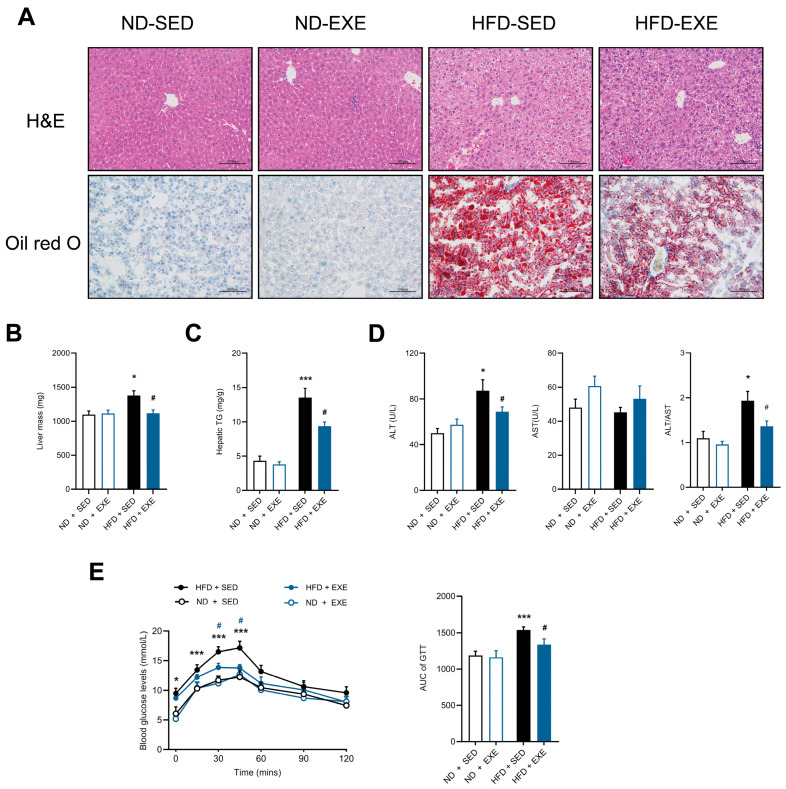
Effects of aerobic exercise on HFD-induced hepatic steatosis and liver injury in mice. (**A**) Representative images of H&E and Oil Red O staining showing hepatic lipid accumulation (scale bar: 100 μm); (**B**) Liver mass. (**C**) Hepatic TG level. (**D**) Serum ALT and AST levels (U/L) and ALT/AST ratio. (**E**) Intraperitoneal GTT with blood glucose levels measured at indicated time points (*n* = 5 per group). Data are presented as mean ± SEM. * *p* < 0.05, *** *p* < 0.001 vs. ND + SED group; # *p* < 0.05 vs. ND + HFD group (two-way ANOVA with Bonferroni post hoc test), *n* = 5 per group.

Histopathological evaluation through H&E and Oil Red O staining demonstrated that 16-week HFD feeding induced characteristic hepatocellular vacuolar degeneration and substantial intrahepatic lipid accumulation (Figure 1A). Subsequent 8-week aerobic exercise intervention effectively ameliorated HFD-induced hepatic steatosis progression and attenuated diet-associated increases in liver wet weight (Figure 1B). Biochemical analysis revealed that aerobic exercise significantly suppressed HFD-elevated hepatic TG content (Figure 1C).

Consistent with clinical features of fatty liver disease, HFD feeding elevated serum ALT levels without affecting AST, and exercise intervention selectively normalized ALT activity (Figure 1D), indicating hepatoprotective effects. GTT further demonstrated that aerobic exercise improved impaired glucose clearance capacity in HFD-fed mice (Figure 1E). Therefore, our study demonstrates that long-term aerobic exercise alleviates high-fat diet-induced hepatic lipid accumulation and liver injury, while also improving glucose metabolism in mice.

### 3.2. Aerobic Exercise Restructures Gut Microbial Composition in High-Fat Diet Mouse Models

HFD and metabolic disorders are often accompanied by alterations in gut microbial community structure. Previous studies have demonstrated that exercise can modulate the composition of gut microbiota in normal mice. In this study, we performed 16S rRNA gene sequencing analysis on fecal samples to evaluate the effects of HFD and exercise intervention on gut microbiota structure.

**Figure 2:**
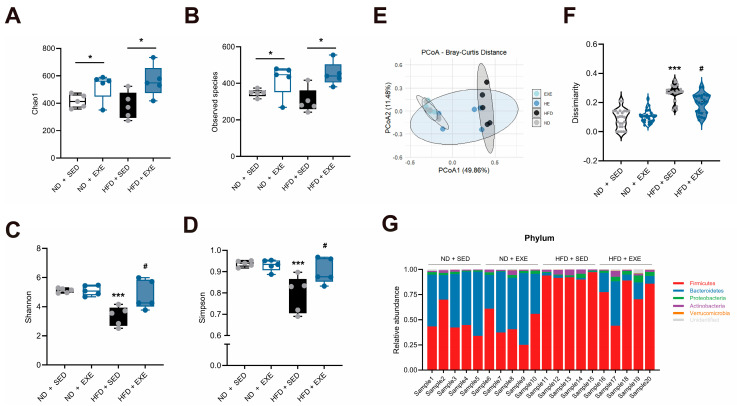
Effects of aerobic exercise on gut microbiota diversity and composition in HFD-fed mice. (**A**) Observed species richness. (**B**–**D**) α-Diversity indices (Chao1, Simpson, and Shannon) at the genus level. ((**A**–**D**), Each data point represents one individual sample) (**E**) Principal coordinate analysis (PCoA) of β-diversity. (**F**) Inter-group dissimilarity (Bray–Curtis distance). (**G**) Taxonomic composition at the phylum level (relative abundance of dominant genera). Data are presented as mean ± SEM. * *p* < 0.05, *** *p* < 0.001 vs. ND + SED group; # *p* < 0.05 vs. ND + HFD group (two-way ANOVA with Bonferroni post hoc test), *n* = 5 per group.

Alpha diversity, which measures species richness and evenness within communities, was analyzed. As shown in Figure 2A,B, long-term aerobic exercise increased both the observed species number and Chao1 index in both HFD-fed and ND mice, indicating that exercise significantly enhanced microbial richness. In contrast, HFD did not significantly affect the overall species count, suggesting that exercise may play a dominant role in regulating gut microbiota structure. Further evaluation of dominant species diversity and evenness using Shannon and Simpson indices (Figure 2C,D) revealed that HFD significantly reduced microbial diversity, while exercise intervention effectively reversed this change and restored diversity levels in HFD-fed mice.

Beta diversity analysis was conducted to examine structural differences in microbial composition between experimental groups. Principal coordinates analysis (PCoA) based on weighted UniFrac distance (Figure 2E) showed that the first and second principal components explained 49.86% and 11.48% of the variation, respectively, indicating significant differences in microbiota structure among groups. PERMANOVA and ANOSIM analyses further confirmed these significant differences (R = 0.566, *p* = 0.001), demonstrating high heterogeneity in microbial composition between treatment groups. To quantify the extent of structural changes, we calculated weighted UniFrac distances relative to the ND-SED group (Figure 2F). Results showed that HFD caused the most significant deviation in microbial structure, but this difference was substantially reduced after 8 weeks of exercise intervention, suggesting that exercise partially restored gut microecological homeostasis. Furthermore, phylum-level comparisons of microbial composition (Figure 2G) demonstrated that HFD significantly altered gut microbiota, while exercise intervention markedly alleviated these changes and promoted microbial restructuring toward normalization. In summary, long-term aerobic exercise effectively ameliorates high-fat diet-induced gut microbiota dysbiosis by enhancing microbial diversity and stability.

### 3.3. Aerobic Exercise-Induced Alterations in Gut Microbial Composition Are Associated with Bile Acid Metabolism

**Figure 3:**
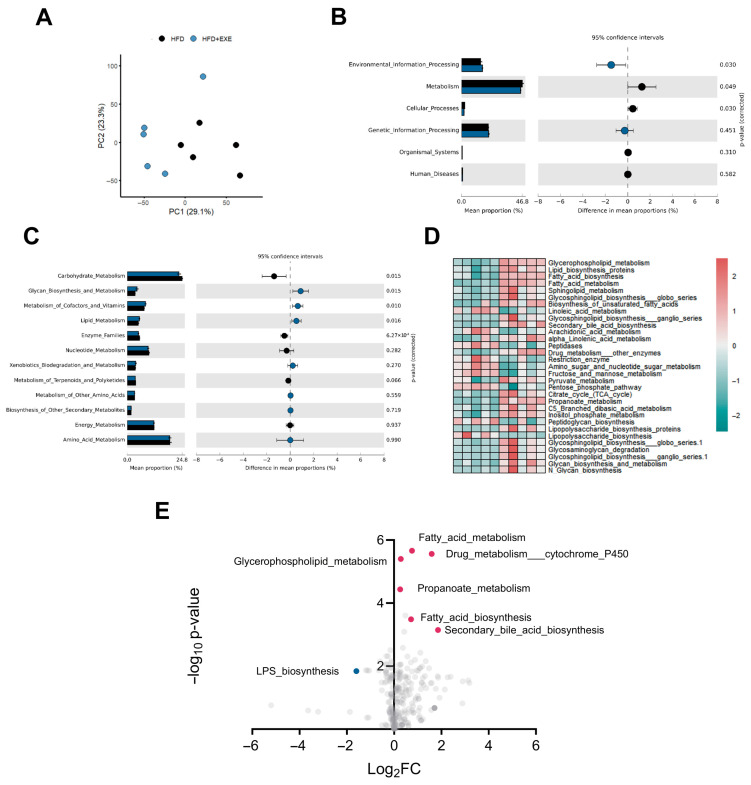
Functional analysis of gut microbiota remodeling by aerobic exercise in HFD-fed mice. (**A**) PCA plot of OTUs. (**B**) KEGG pathway enrichment (Level 1 categories). (**C**) Metabolic pathway alterations (KEGG Level 2). (**D**) Heatmap of differentially enriched KEGG pathways (Level 3) clustered by the top four Level 2 metabolic categories. (**E**) Volcano plot highlighting significantly altered KEGG pathways (Level 3) between HFD + SED and HFD + EXE groups, the blue dot means downregulate, and red means upregulate. *n* = 5 per group.

To further elucidate the role of gut microbiota alterations in exercise-mediated amelioration of HFD induced hepatic steatosis, we performed KEGG functional annotation analysis on intestinal microbiota from HFD sedentary (HFD + SED) and HFD exercise (HFD + EXE) groups. PCA of the annotated functions revealed distinct clustering patterns (PC1: 29.1%, PC2: 23.3%), indicating significant functional divergence between groups (Figure 3A). Comparative analysis identified major differences in KEGG level-1 categories including Environmental Information Processing, Metabolism, and Cellular Processes, suggesting exercise may modulate microbial functions related to intestinal environmental sensing and metabolic regulation (Figure 3B). Focusing on metabolic disorders, we specifically analyzed Metabolism-associated pathways and found that exercise significantly altered multiple metabolic functions: Carbohydrate metabolism (*p* = 0.015), Glycan biosynthesis and metabolism (*p* = 0.015), Cofactors and vitamins metabolism (*p* = 0.010), Lipid metabolism (*p* = 0.016), and Enzyme families (*p* < 0.001) (Figure 3C). Heatmap visualization demonstrated these differentially regulated pathways (Figure 3D). Notably, exercise intervention markedly upregulated several lipid metabolism-related pathways (e.g., lipid metabolism, glycerophospholipid metabolism, and fatty acid metabolism) in HFD-fed mice. Volcano plot analysis further revealed the directional changes, showing significant upregulation of multiple lipid metabolic pathways alongside decreased LPS biosynthesis in exercised mice. Most prominently, secondary bile acid biosynthesis pathways showed the most substantial upregulation among all altered pathways (Figure 3E). These results indicate that long-term exercise alters the gut microbiota structure in high-fat diet-fed mice, and the observed differences in microbial abundance are primarily associated with the regulation of lipid metabolism and secondary bile acid-related processes.

**Figure 4:**
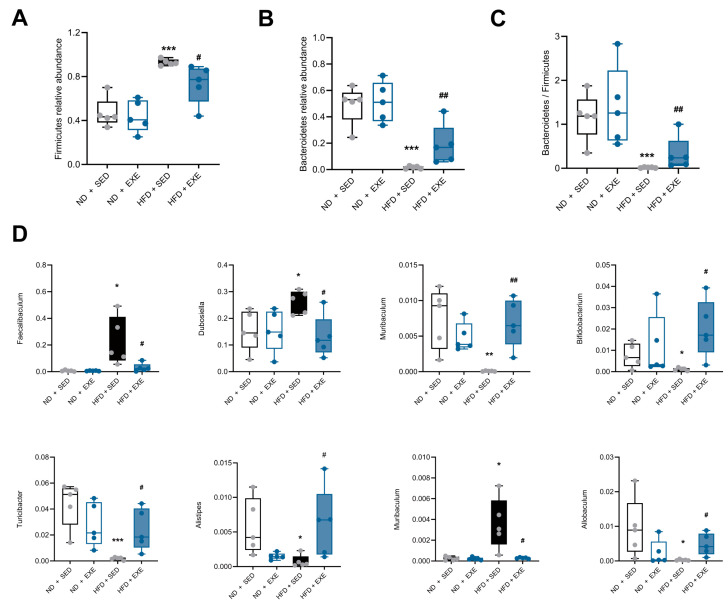
Aerobic exercise restores gut microbiota composition in HFD-fed mice. (**A**) Relative abundance of Firmicutes. (**B**) Relative abundance of Bacteroidetes. (**C**) Firmicutes and Bacteroidetes ratio (**D**) other genus relative abundance based on the genus profile in the four groups (Each data point represents one individual sample). Data are presented as mean ± SEM. * *p* < 0.05, *** *p* < 0.001 vs. ND + SED group; # *p* < 0.05, ## *p* < 0.01 vs. ND + HFD group (two-way ANOVA with Bonferroni post hoc test), *n* = 5 per group.

To validate these findings, we further analyzed compositional changes of gut microbiota at the phylum level. The dysregulation of two dominant phyla, *Firmicutes* and *Bacteroidetes*, is commonly observed in HFD and metabolic disorders. Our results demonstrated that HFD significantly increased the relative abundance of *Firmicutes* (*p* < 0.005), while exercise intervention effectively reversed this trend (Figure 4A). Conversely, *Bacteroidetes* showed significant reduction in the HFD group (*p* < 0.005), which was restored after exercise (Figure 4B). Consequently, the *Bacteroidetes*/*Firmicutes* (B/F) ratio improved following exercise intervention (Figure 4C), further supporting the restoration of microbial structure. At the species level, we identified multiple bacterial species associated with lipid and bile acid metabolism that were dysregulated by HFD, including increased abundance of *Faecalibaculum*, *Dubosiella*, and *Muribaculum*, along with decreased *Bifidobacterium*, *Turicibacter*, *Allobaculum*, *Alistipes*, and *Ileibacterium*. Long-term exercise intervention effectively normalized these microbial alterations (Figure 4D).

In conclusion, our findings demonstrate that exercise restores high-fat diet-induced alterations in gut microbiota composition.

### 3.4. Aerobic Exercise Restructures the Hepatic Bile Acid Pool in High-Fat Diet-Fed Mice

**Figure 5:**
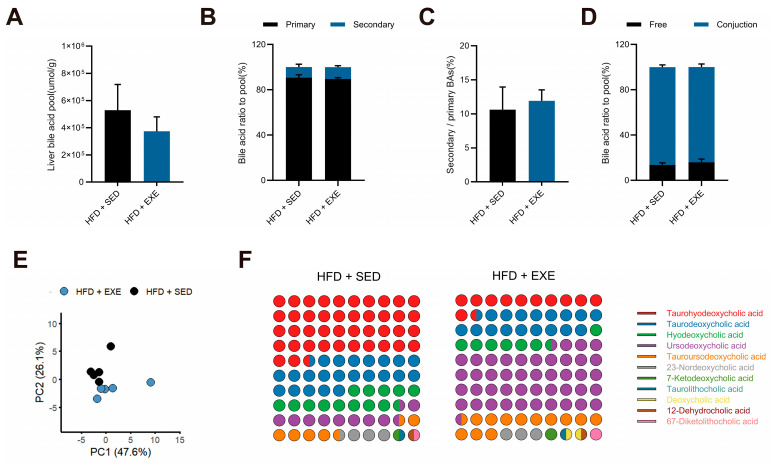
Impact of aerobic exercise on the hepatic bile acid pool in HFD-fed mice. (**A**) Total hepatic bile acid content. (**B**) Composition of primary vs. secondary bile acids. (**C**) Free-to-conjugated bile acid ratio. (**D**) Free and conjunct bile acid ratio in total hepatic bile acid pool. (**E**) PCA of hepatic bile acid profiles. (**F**) Relative abundance of individual secondary bile acids in hepatic bile acid pool. *n* = 5 per group.

Based on the differential results from microbiota functional analysis, we next used the HFD model as a reference to investigate the effects of aerobic exercise in HFD-fed mice. We conducted targeted bile acid metabolomics analysis on liver tissues from high-fat diet sedentary (HFD + SED) and aerobic exercise intervention (HFD + EXE) groups, covering 41 common bile acid species. Initial analysis of total hepatic bile acid levels revealed no significant differences between groups (Figure 5A). Further examination of the relative proportions and absolute concentrations of primary versus secondary bile acids in the bile acid pool similarly showed no significant alterations (Figure 5B,C), suggesting that exercise intervention had limited effects on overall bile acid synthesis capacity in HFD-fed mice. Additionally, comparative analysis of unconjugated versus conjugated bile acid levels demonstrated no significant changes between groups (Figure 5D), indicating that exercise did not substantially influence bile acid conjugation processes.

To further elucidate changes in the proportional composition of individual bile acids within the total bile acid pool, we performed PCA to compare the overall bile acid profiles between groups. The analysis revealed that PC1 and PC2 accounted for 47.6% and 26.1% of the total variance, respectively, indicating substantial differences in hepatic bile acid composition between the two groups (Figure 5E). Given the preliminary findings suggesting potential alterations in secondary bile acid metabolism, we specifically focused on relative abundance changes within the secondary bile acid pool. The results demonstrated significant fluctuations in the proportions of several secondary bile acids, with particularly notable changes observed in ursodeoxycholic acid (UDCA) and THDCA (Figure 5F). These findings suggest that long-term exercise intervention alters the compositional distribution of secondary bile acids in the HFD-fed mouse liver without affecting the overall size of the bile acid pool.

### 3.5. Aerobic Exercise Modulates Gut Microbiota to Enhance CDCA-Derived and Reduce CA-Derived Secondary Bile Acid Metabolism

**Figure 6:**
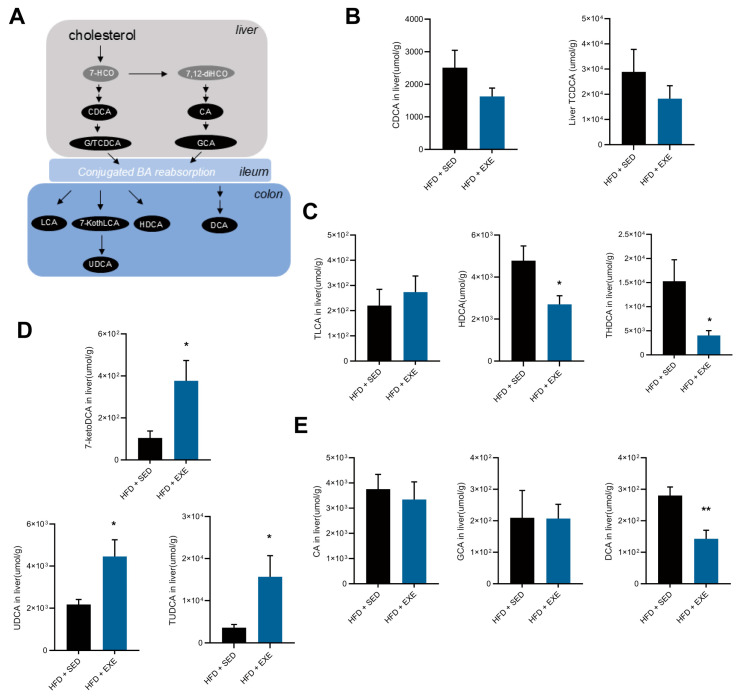
Effects of aerobic exercise on hepatic TUDCA, DCA, and their precursor levels in HFD-fed mice. (**A**) Biosynthetic pathways of UDCA and DCA from primary bile acids, arrows depict the biosynthetic direction from substrates to products (**B**) Hepatic levels of free and conjugated CDCA. (**C**) The levels of major secondary bile acids generated from CDCA (chenodeoxycholic acid) as a precursor. (**D**) The level of UDCA and its precursors, derivative in total hepatic bile acid pool. (**E**) Relative abundance of the precursor of DCA and DCA in the liver. Compare with HFD + SED group (*t*-test), * *p* < 0.05, ** *p* < 0.01, *n* = 5 per group.

Building upon the observed effects of aerobic exercise on hepatic secondary bile acid composition, this study focused on two critical bile acid species: UDCA and THDCA, along with their respective metabolic derivative TUDCA and precursors and hyodeoxycholic acid (HDCA) (Figure 6A). These secondary bile acids share chenodeoxycholic acid (CDCA) as their common biosynthetic precursor. To determine whether the observed changes in TUDCA and THDCA levels resulted from gut microbial modification rather than alterations in endogenous synthesis, we first compared hepatic levels of free and conjugated CDCA between experimental groups. The absence of significant intergroup differences in free and conjugated CDCA levels (Figure 6B) suggested that the observed variations in TUDCA and THDCA were not attributable to differential CDCA synthesis capacity. Subsequent analysis of other major microbial-derived secondary bile acids revealed that while conjugated lithocholic acid (TLCA) remained unchanged, both free HDCA and its conjugated form (THDCA) showed significant reductions (Figure 6C). Furthermore, comparative analysis demonstrated marked increase in UDCA, its conjugated form (TUDCA), and their precursor 7-ketodeoxycholic acid (7-ketoDCA) (Figure 6D), indicating that the alterations in TUDCA levels primarily reflected changes in microbial hydroxylation efficiency rather than hepatic synthesis. Investigation of an alternative secondary bile acid pathway involving the FXR antagonist deoxycholic acid (DCA) showed significant reductions in DCA levels without corresponding changes in its precursors cholic acid (CA) or conjugated glycocholic acid (GCA) (Figure 6E), confirming that the decreased DCA levels did not result from enhanced upstream synthesis. Collectively, these findings demonstrate that the transformation capacity of gut microbiota for primary bile acids serves as the principal determinant of the observed changes in hepatic secondary bile acid profiles.

**Figure 7:**
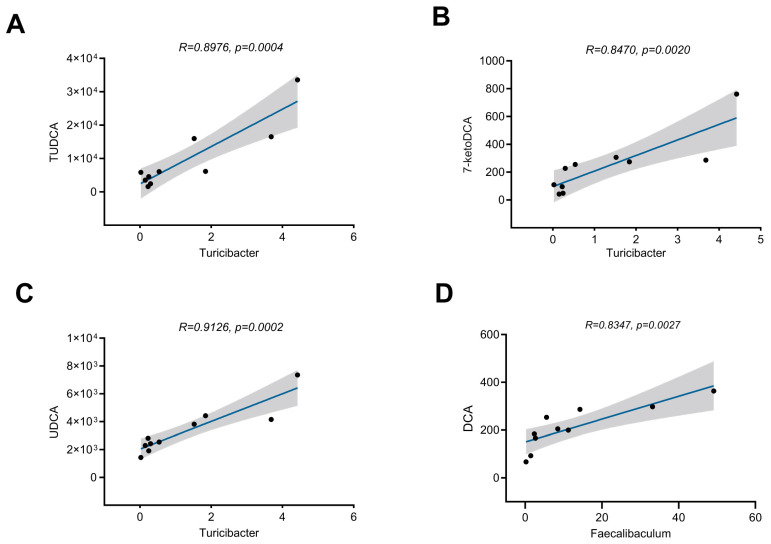
Correlation analysis between secondary bile acid levels and microbiota abundance. (**A**–**C**) Spearman correlation between hepatic TUDCA (**A**), 7-ketoDCA (**B**), UDCA (**C**) levels and Turicibacter abundance. (**D**) Correlation between hepatic DCA levels and *Faecalibaculum* abundance.

To validate this hypothesis, we performed Spearman correlation analysis between differential bile acids and microbial taxa. The results revealed significant positive correlations between: TUDCA levels and *Turicibacter* abundance (*r* = 0.8476, *p* = 0.0004, Figure 7A); UDCA levels and *Turicibacter* abundance (*r* = 0.9126, *p* = 0.0002, Figure 7B); 7-ketoDCA (TUDCA precursor) levels and *Turicibacter* abundance (*r* = 0.8470, *p* = 0.002, Figure 7C); and hepatic DCA levels and *Faecalibaculum* abundance (*r* = 0.8347, *p* = 0.0027, Figure 7D). These findings collectively demonstrate that the observed changes in hepatic secondary bile acid (particularly TUDCA and DCA) abundance induced by long-term exercise are likely unrelated to the synthesis of their precursor primary bile acids, but are instead associated with gut microbiota (e.g., *Turicibacter* and *Faecalibaculum*)-mediated modification and synthesis of secondary bile acids within the intestinal tract.

### 3.6. Aerobic Exercise Attenuates Hepatic Lipid Metabolism via Secondary Bile Acid-Mediated ERS Alleviation and FXR Activation

Secondary bile acids, generated through enzymatic modification of primary bile acids by gut microbiota, participate in multiple signaling pathways to regulate hepatocyte function. Notably, TUDCA, a well-characterized ERS inhibitor, has attracted significant attention in lipid metabolism disorders. Given the close association between hepatic steatosis and ERS, this study further investigated the effects of aerobic exercise intervention on the expression of ERS-related molecules in the liver.

**Figure 8:**
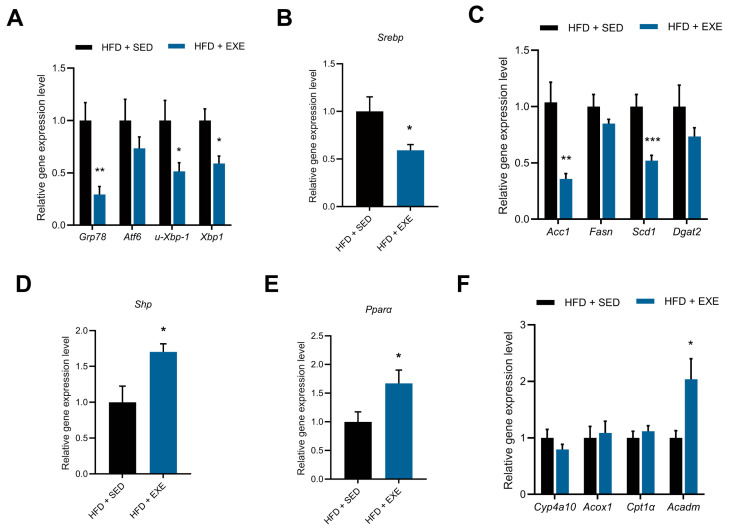
Aerobic exercise regulates hepatic lipid metabolism gene expression through ER stress and FXR-SHP signaling pathway. (**A**) Hepatic ER stress marker gene expression. (**B**,**C**) Lipogenic pathway genes: (**B**) Srebp1 and (**C**) its downstream targets. (**D**) FXR activation marker Shp. (**E**,**F**) Fatty acid oxidation genes: (**E**) Ppara and (**F**) its targets. Compare with HFD + SED group (*t*-test), * *p* < 0.05, ** *p* < 0.01, *** *p* < 0.005, *n* = 5 per group.

The results demonstrated that long-term aerobic exercise significantly reduced the expression of GRP78 (Glucose-Regulated Protein 78), a key ERS marker, and markedly suppressed both spliced (XBP1s) and unspliced (XBP1u) forms of XBP1 (Figure 8A), indicating effective alleviation of HFD-induced hepatic ERS. To investigate the metabolic consequences of ERS attenuation, we analyzed the expression of sterol regulatory element-binding protein 1 (Srebp1), a downstream lipid metabolism regulator of ERS, and observed significant downregulation (Figure 8B). Concurrently, expression of TG synthesis-related genes acetyl-CoA carboxylase 1 (Acc1) and stearoyl-CoA desaturase 1 (Scd1) downstream of Srebp were significantly inhibited, while fatty acid synthase (Fasn) and Diacylglycerol O-acyltransferase 2 (Dgat2) remained unchanged (Figure 8C). Besides TUDCA, another crucial secondary bile acid DCA was confirmed to activate the nuclear bile acid receptor FXR and induce expression of its downstream transcriptional factor SHP. Our data revealed significantly upregulated hepatic SHP expression in exercised mice (Figure 8D), suggesting FXR pathway activation. Further examination showed increased expression of peroxisome proliferator-activated receptor alpha (Pparα), a key transcriptional factor for fatty acid oxidation (Figure 8E), and its downstream target gene medium-chain acyl-CoA dehydrogenase (Acadm), whereas genes related to ω-oxidation and peroxisomal fatty acid oxidation showed no significant changes (Figure 8F), indicating exercise primarily enhances mitochondrial fatty acid oxidation to improve hepatic lipid metabolism.

In conclusion, the ameliorative effect of exercise on hepatic steatosis may be associated with potential pathways mediated by secondary bile acids, including the alleviation of endoplasmic reticulum stress and alterations in the expression of lipid metabolism genes following activation of the FXR signaling pathway.

## 4. Discussion

Unhealthy dietary patterns, particularly high-fat diets, represent a key contributing factor to hepatic steatosis. Dietary components promote the proliferation of specific dominant gut microbiota while generating metabolite, such as secondary bile acids to regulate host metabolic function [22]. External stimuli modulate microbial bile acid transformation to regulate hepatic metabolism [23]. Physical activity influences intestinal homeostasis and shaping microbial colonization and growth [24,25]. While exercise is known to ameliorate hepatic steatosis, existing research has predominantly focused on its direct effects on hepatic or metabolic tissues, with limited understanding of enterohepatic communication. In this study, integrated 16S rRNA amplicon sequencing and targeted metabolomics revealed that: (1) Aerobic exercise modifies the gut microbial structure in HFD-fed mice, and the functional profiles of these differentially abundant microbes are associated with lipid metabolism and secondary bile acid synthesis; (2) Aerobic exercise alters the compositional distribution of secondary bile acids in the liver. Key secondary bile acids (TUDCA, DCA) involved in this process are associated with changes in gut microbiota abundance and may participate in regulating the expression of hepatic lipid metabolism genes.

### 4.1. Effects of Long-Term Exercise Training on Gut Microbiota Diversity in HFD-Fed Mice

Microbial diversity serves as a crucial indicator of microbial community functionality and stability, representing a key biomarker for health status assessment [26]. Elite athletes exhibit higher gut microbiota diversity [27]. Exercise training has been shown to modulate the abundance of dominant bacterial species and secondary metabolite levels in both healthy and obese individuals [18,28]. Reduced microbial richness and diversity are associated with various metabolic abnormalities, including elevated adiposity, insulin resistance, and dyslipidemia [29]. The present study demonstrates that long-term aerobic exercise increases gut microbial richness (assessed by species count and Chao1 index) in both HFD-ND mice, concomitant with improvements in insulin sensitivity and lipid profiles. These findings align with previous research indicating that exercise enhances microbial quantity and diversity in murine models [27,30,31]. In a MAFLD mouse model, exercise was found to optimize gut barrier integrity and restructure gut microbiota composition, thereby ameliorating hepatic steatosis and hyperlipidemia [32].

The Simpson and Shannon indices reflect species evenness within microbial communities. In the present study, HFD-fed mice exhibited reduced microbial evenness, while exercise intervention effectively restructured the gut microbiota composition and improved species evenness. This improvement was primarily driven by specific alterations in the relative abundance of dominant bacterial phyla, particularly *Bacteroidetes* and *Firmicutes*. The ratio of *Bacteroidetes* to *Firmicutes* has been consistently associated with obesity and metabolic disorders, as it directly influences dietary nutrient digestion and absorption efficiency [33]. Notably, these findings align with previous reports demonstrating that two weeks of moderate-intensity exercise training increases *Bacteroidetes* abundance and elevates the *Bacteroidetes*-to-*Firmicutes* ratio in both obese/insulin-resistant individuals and HFD-fed animal models [34,35,36]. Importantly, these dominant bacterial phyla have been extensively documented to participate in bile acid modification processes, a finding further corroborated by subsequent analyses in this study [28].

This study also identified several exercise-modulated gut microbiota taxa altered by HFD, including *Turicibacter*, *Faecalibaculum*, *Dubosiella*, *Bifidobacterium*, *Allobaculum*, *Alistipes*, *Muribaculum*, and *Ileibacterium*. Functional characterization revealed that: (1) *Bifidobacterium* and *Turicibacter* ameliorate hepatic steatosis through acetate production (*Bifidobacterium*-derived acetate particularly alleviates HFD-induced hepatocellular vacuolation) [37]; (2) *Dubosiella* modulates hepatic TG synthesis [38]; (3) *Faecalibaculum* abundance positively correlates with liver injury [39]; and (4) *Allobaculum*, *Alistipes*, *Muribaculum*, and *Ileibacterium* exhibit hepatoprotective effects against lipid accumulation [40,41,42,43].

### 4.2. Effects of Long-Term Exercise Training on Liver Bile Acid Pool in HFD-Fed Mice

Bile acids represent the most extensively studied molecules in primary signaling mediators between gut microbiota and the liver [44]. Primary bile acids are synthesized from cholesterol in the liver; secondary bile acids are formed through microbial modifications of primary bile acids in the gut [23]. Aerobic exercise increased total bile acid levels in both fecal and blood samples of HFD mice [45,46,47]. However, changes in total bile acid levels in blood and liver do not always align [48]. As the central regulatory organ for bile acid metabolism, the liver maintains bile acid pool homeostasis through dynamic balancing mechanisms rather than unidirectional modulation [49].

We employed PCA to characterize exercise-induced alterations in bile acid diversity in HFD-fed mice liver. The results revealed structural changes in secondary bile acid composition, with the most pronounced variations observed in the TUDCA and DCA ratio. Notably, this study is the first to demonstrate that chronic exercise intervention elevates hepatic TUDCA levels in HFD mice. Previous studies have also confirmed TUDCA’s therapeutic potential against hepatic steatosis [50]. Our findings showed increased hepatic TUDCA without corresponding changes in its precursor CDCA, coupled with reduced parallel-pathway secondary bile acids of intestinal origin, suggesting gut microbiota-mediated modification rather than hepatic synthesis as the underlying mechanism. This observation also explains the absence of overall secondary bile acid level changes in prior experiments. Regarding DCA dynamics, this aligns with clinical evidence showing exercise training reduces serum DCA levels in diabetic patients [51].

This study also identified two key microbiota species changed by exercise: *Turicibacter* and *Faecalibaculum*. Previous research confirmed that changes in *Turicibacter* abundance were positively correlated with obesity-related lipid metabolism characteristics, and *Turicibacter* colonization altered host serum lipid metabolome and mice TG levels [52]. Furthermore, the study found that *Turicibacter* species can effectively regulate bile acid formation, particularly UDCA, the unconjugated form of TUDCA [53]. HPLC/MS-MS in vitro validation experiments revealed that *Turicibacter* possesses 7α-hydroxysteroid dehydrogenase activity, which can increase the abundance of 7-ketoDCA, a TUDCA precursor [54]. The results showed elevated hepatic 7-ketoDCA levels, with significant positive correlations between 7-ketoDCA, UDCA, TUDCA and *Turicibacter*. Therefore, the exercise-induced increase in hepatic TUDCA levels may result from *Turicibacter* activity in the gut. The other differential microbiota species was *Faecalibaculum*. Studies have shown that *Faecalibaculum* is positively correlated with serum lipids and MAFLD progression [55]. Multiple studies have demonstrated concurrent increases in intestinal DCA levels and *Faecalibaculum* abundance, and our study also found a positive correlation between changes in *Faecalibaculum* abundance and DCA levels [39,43]. This suggests that *Faecalibaculum* may be involved in regulating DCA levels, although the specific mechanisms require further clarification.

### 4.3. Potential Molecular Mechanisms of Exercise-Induced Hepatic Steatosis Amelioration

Abnormal hepatic lipid accumulation frequently co-occurs with insulin resistance and is associated with ERS in hepatocytes [56]. The unfolded protein response, particularly the Inositol requiring enzyme 1 alpha (IRE1α)-mediated pathway regulates lipid synthesis [56]. During hepatic steatosis development, elevated GRP78 expression and increased formation of XBP1 mRNA splicing variants serve as hallmark indicators of ERS [57]. The amelioration of hepatic ER stress constitutes a crucial mechanism through which exercise improves lipid metabolism disorders. Our study identified exercise-induced elevation of TUDCA, a potent ERS inhibitor, suggesting that exercise may alleviate hepatic lipid accumulation by enhancing TUDCA-mediated ERS suppression. Subsequent experiments demonstrated that exercise intervention effectively reduced hepatic ERS in high-fat diet-fed mice, downregulating the transcriptional activity of SREBP, a key lipid synthesis regulator downstream of IRE1α. Concurrently, expression of ACC1 and SCD1—critical enzymes for TG synthesis and confirmed direct targets of SREBP—was significantly inhibited [58]. Existing research has established that oral TUDCA administration exerts hepatoprotective effects in aging mice by alleviating ER stress [48]. In vitro studies have also reported that TUDCA can suppress endoplasmic reticulum stress and the upregulation of SREBP1 in HepG2 cells exposed to high fructose conditions [59]. These findings collectively indicate that TUDCA-mediated ERS alleviation represents a plausible pathway through which exercise ameliorates hepatic steatosis.

The farnesoid X receptor (FXR) present in hepatocytes serves as a primary receptor for bile acid [60]. FXR activation regulates key genes involved in both bile acid synthesis and lipid metabolism, thereby ameliorating hepatic lipid accumulation [60,61]. Upon binding with bile acids, FXR enhances the transcription of its downstream effector SHP, thereby modulating hepatic lipid metabolism by regulating metabolic genes such as Pparα [43]. Although no alterations were detected in CDCA (the primary FXR agonist) [48], we still observed elevated FXR activation and subsequent SHP upregulation. Previous study found that reduced DCA levels can modulate FXR activation to alleviate hepatic steatosis [48], which suggest decreased DCA in our study maybe play the role. Our results showed FXR activation, which aligns with previous studies demonstrating that exercise promotes FXR cleavage and subsequent downstream signaling cascades in the liver [62]. In this study, exercise intervention upregulated both Pparα and its downstream Acadm levels in high-fat diet-fed mice. These results align with other study which showed that exercise increase hepatic Acadm expression and boost mitochondrial fatty acid oxidation capacity [63]. Therefore, FXR activation-induced fatty acid oxidation is related to exercise-mediated amelioration of hepatic steatosis.

### 4.4. Limitations and Future Perspectives

Although this study elucidates the mechanism by which exercise alleviates hepatic steatosis from the perspective of the gut-liver axis, several limitations remain. First, we cannot definitively establish whether gut microbiota is essential for exercise-mediated amelioration of hepatic steatosis—this would require verification through microbiota transplantation or antibiotic treatment experiments. Secondly, although the current sample size allowed for statistical evaluation in partial experiments, increasing the number of biological replicates in follow-up studies would enhance the statistical power and provide more compelling evidence for our conclusions. Additionally, while we have measured the overall changes in bile acid levels in the liver, profiling bile acid alterations in both the intestine and bloodstream would provide a more comprehensive understanding of the role of secondary bile acids within the enterohepatic circulation.

## 5. Conclusions

**Figure 9:**
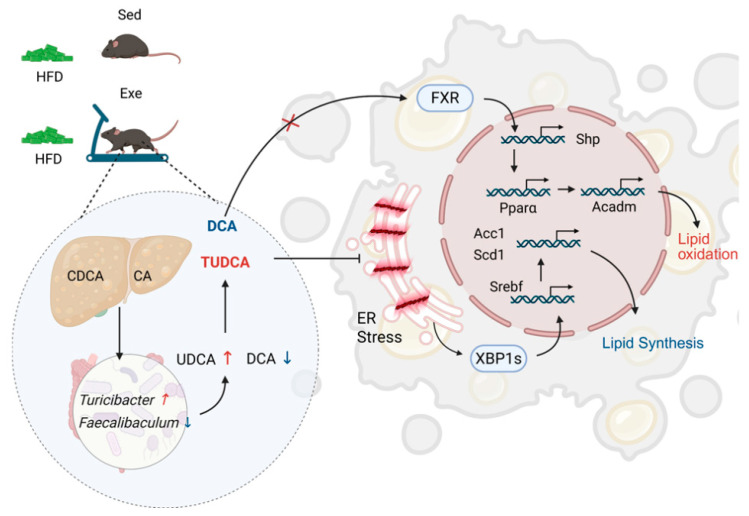
Integrated gut-liver axis mechanisms by which aerobic exercise alleviates hepatic steatosis in mice. The black arrows indicate the fate of metabolites and proteins, the colored arrows represent the direction of abundance changes, and the vertical lines denote inhibition.

Our findings demonstrate that aerobic exercise changes the gut microbiota composition and enhances its diversity in high-fat diet-fed mice. The observed alterations in the abundance of hepatic secondary bile acids (TUDCA and DCA) were associated with these microbial changes. Furthermore, the exercise-induced modulation of TUDCA and DCA may contribute to the regulation of hepatic lipid metabolism gene expression (Figure 9).

## Data Availability

The original contributions presented in the study are included in the article; further inquiries can be directed to the corresponding author.

## Abbreviations

HFD	high-fat diet
ND	Normal diet
ND-SED	Normal diet with sedentary
ND-EXE	Normal diet with exercise
HFD-SED	High fatty diet with sedentary
HFD-EXE	High fatty diet with exercise
MAFLD	Metabolic dysfunction-associated fatty liver disease
TG	Triglyceride
ER	Endoplasmic reticulum
GTT	Glucose tolerance test
ALT	Alanine Aminotransferase
AST	Aspartate Aminotransferase
PCA	Principal component analysis
PCoA	Principal coordinates analysis
OTUs	Operational Taxonomic Units
KEGG	Kyoto Encyclopedia of Genes and Genomes
GRP78	Glucose-Regulated Protein 78
FXR	Farnesoid X receptor
Srebp	Sterol Regulatory Element-Binding Protein
Acc1	Acetyl-CoA carboxylase 1
Scd1	Stearoyl-CoA desaturase 1
Xbp1s/u	X-box binding protein 1 spliced/ unspliced
SHP	Short heterodimer partner
Pparα	Peroxisome proliferator-activated receptor alpha
Acadm	Medium-chain acyl-CoA dehydrogenase
IRE1α	Inositol requiring enzyme 1 alpha
DGAT2	Diacylglycerol O-Acyltransferase 2
FASN	Fatty Acid Synthase
TUDCA	Tauroursodeoxycholic acid
UDCA	Ursodeoxycholic acid
THDCA	Taurohyodeoxycholic
CDCA	Chenodeoxycholic acid
DCA	Deoxycholic acid
TDCA	Taurodeoxycholic acid
CA	Cholic Acid
GCA	Glycocholic acid
7-ketoDCA	7-ketodeoxycholic acid (7-ketoDCA)
SCFA	Short-chain fatty acid
